# Natural behaviours after guided growth for idiopathic genu valgum correction: comparison between percutaneous transphyseal screw and tension-band plate

**DOI:** 10.1186/s12891-022-05996-1

**Published:** 2022-12-03

**Authors:** Byoung Kyu Park, Hyun Woo Kim, Hoon Park, Seung-Kyu Lee, Kun-Bo Park

**Affiliations:** 1 Department of Orthopaedic Surgery, Inje University Haeundae Paik Hospital, Busan, South Korea; 2 Division of Pediatric Orthopaedic Surgery, Severance Children’s Hospital, Yonsei University College of Medicine, 50-1 Yonsei-ro, Seodaemun-gu, 03722 Seoul, South Korea; 3 Department of Orthopaedic Surgery, Gangnam Severance Hospital, Yonsei University College of Medicine, Seoul, South Korea

**Keywords:** Genu valgum, Guided growth, Percutaneous transphyseal screw, Tension-band plate, Rebound, Overcorrection

## Abstract

**Background:**

Percutaneous epiphysiodesis using a transphyseal screw (PETS) or tension-band plating (TBP) has shown favourable correction results; however, the physeal behaviours in terms of rebound, stable correction, or overcorrection after guided growth have not been completely understood. In patients with idiopathic genu valgum, we therefore asked: (1) How is the correction maintained after implant removal of guided growth? (2) Is there any difference in the natural behaviours after PETS or TBP removal at the femur and tibia?

**Methods:**

We retrospectively reviewed 73 skeletally immature limbs with idiopathic genu valgum treated with PETS or TBP. PETS was performed in 23 distal femurs and 13 proximal tibias, and TBP was performed in 27 distal femurs and ten proximal tibias. Mechanical axis deviation (MAD), mechanical lateral distal femoral angle (mLDFA), and mechanical medial proximal tibial angle were measured at pre-correction, implant removal, and final follow-up. Changes of ≤ 3° in mechanical angles after implant removal were considered stable. Comparisons between the implant, anatomical site, and existence of rebound were performed.

**Results:**

The mean MAD improved from − 18.8 mm to 11.3 mm at implant removal and decreased to -0.2 mm at the final follow-up. At the final follow-up, 39 limbs (53.4%) remained stable and only 12 (16.4%) were overcorrected. However, 22 limbs (30.1%) showed rebound. TBP was more common, and the correction period was longer in the rebound group (*p* < 0.001 and 0.013, respectively). In femurs treated with PETS, the mean mLDFA increased from 86.9° at implant removal to 88.4° at the final follow-up (*p* = 0.031), demonstrating overcorrection. However, a significant rebound from 89.7° to 87.1° was noted at the femur in the TBP group (*p* < 0.001). The correction of the proximal tibia did not change after implant removal.

**Conclusion:**

The rebound was more common than overcorrection after guided growth; however, approximately half the cases demonstrated stable correction. The overcorrection occurred after PETS in the distal femur, while cases with TBP had a higher probability of rebound. The proximal tibia was stable after implant removal. The subsequent physeal behaviours after each implant removal should be considered in the guided growth.

## Background

Most genu valgum in young children resolve spontaneously; however, deformities beyond the physiological limits can lead to cosmetic problems and produce an uneven mechanical load to the lateral compartment of the knee joint and instability of the patellofemoral joint [1–6]. The genu valgum in skeletally immature patients could be corrected by simple and minimally invasive guided growth such as permanent or temporary hemiepiphysiodesis. Temporary hemiepiphysiodesis has the advantage of being reversible and less risk of permanent growth arrest [7]. It provides gradual deformity correction while the implant is fixed with the asymmetrical suppression of target physis [8], and the suppressed growth can resume when the fixation is removed. After stapling for guided growth [9], percutaneous epiphysiodesis using a transphyseal screw (PETS) and tension-band plate (TBP) have been introduced and gained widespread popularity [10, 11].

Previous studies have shown high success rates of temporary hemiepiphysiodesis using PETS or TBP to correct genu valgum [3–5, 12–15]. Two previous studies reported more rapid correction with PETS than with TBP [4, 5], and this depends on the direct penetration of the physis. The different mechanisms between PETS and TBP for inhibition of growth may also affect the physeal behaviour even after the guided growth implant is removed [2, 4, 15]. However, there is a paucity of studies regarding physeal behaviours after implant removal.

The physeal behaviours that follow removing the guided growth implant make it difficult to determine when the implant should be removed because of unpredictable changes in coronal alignment [2, 8, 12, 15–18]. In terms of rebound, stable correction, and overcorrection, it remains unclear whether PETS and TBP show a difference in their associated physeal behaviours. For analysis of the differences in the physeal behaviours between the two implants, patients with a single etiology and sufficient growing period after implant removal should be compared. In patients with idiopathic genu valgum, we therefore asked: (1) How is the correction maintained after implant removal of guided growth? (2) Is there any difference in the natural behaviours after PETS or TBP removal at the femur and tibia?

## Methods

### Participants

**Fig. 1:**
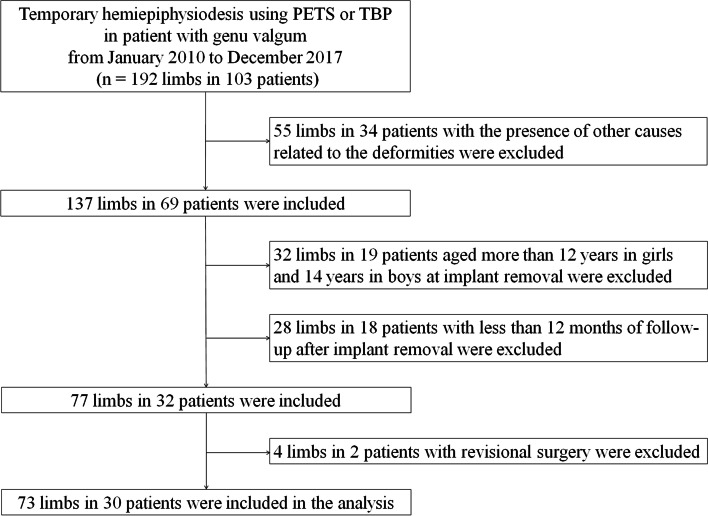
Flow diagram of the inclusion and exclusion process

Our hospital’s Institutional Review Board approved this retrospective study (IRB No. 4-2021-1376). We reviewed the medical records and radiographs of 103 patients with genu valgum who had undergone hemiepiphysiodesis utilizing PETS or TBP in our tertiary children’s hospital between 2010 and 2017. The inclusion criteria were (1) idiopathic genu valgum without any other cause for deformities such as trauma, skeletal dysplasia, endocrinopathy, and neuromuscular disease; (2) follow-up until skeletal maturation. The exclusion criteria were as follows: (1) patients aged > 12 years in girls and 14 years in boys at the time of implant removal; (2) radiologic follow-up < 12 months after implant removal; (3) revisional surgery during the follow-up (Fig. 1). Two patients who had undergone the revisional surgery kept the implant for more than two years because of follow-up loss and personal problems. Finally, 73 limbs in 30 patients were included.

Data collected for the study included patient age at the time of surgery, sex, weight, height, body mass index (BMI), implant type, and operation site. There were 12 boys and 18 girls, and the mean age was 11.2 ± 1.7 years at the time of implant insertion and 11.9 ± 1.8 years at the time of removal (Table 1). PETS was performed in 23 distal femurs and 13 proximal tibias, and TBP was performed in 27 distal femurs and ten proximal tibias.

**Table 1:** Summary of patients’ data

Parameters	Values
Age (years)	11.2 ± 1.7
Male:Female (no. of patients)	12:18
Weight (kg)	55.2 ± 15.1
Height (cm)	151.4 ± 12.1
BMI (kg/m^2^)	23.6 ± 4.4
Operation details (no. of limbs)	
Femur vs. Tibia	50 vs. 23
PETS vs. TBP	36 vs. 37

*BMI* body mass index, *PETS* percutaneous epiphysiodesis using a transphyseal screw, *TBP* tension-band plating

### Surgical technique

Each implant’s characteristics, such as the correction rate, scar size, penetration of the growth plate, and feasibility considering the height of the epiphysis, were explained in detail before surgery. The choice of implant was made based on the informed consent provided by the patients and their parents.

PETS was performed as described by Metaizeau et al. [10]. A stab incision over the distal femoral or proximal tibial diaphysis was made, and a guidewire was inserted obliquely across the metaphysis and into the epiphysis so that the physis crossed a zone comprising one-third to one-fourth of the physeal width. On the lateral view, care was taken to ensure that the guidewire crossed the midpoint of the physis. A retrograde guide pin insertion technique was used at the distal femur. After confirming the guidewire’s position under an image intensifier, a partially threaded cannulated screws (AO Cannulated Screw, Synthes, West Chester, PA) was inserted percutaneously. The screw length was selected to adequately gain purchase on the epiphysis with at least three threads while ensuring that the screw head would not be buried within the periosteum, as visualized by an image intensifier.

TBP with two cannulated screws was placed spanning the growth plate (Orthofix, McKinney, TX) as previously described [11]. Through a 3–4 cm incision, each plate was placed in a submuscular or subfascial position, with care taken to preserve the periosteum. Fluoroscopy was used to verify satisfactory plate placement.

All patients were allowed to bear their full weight on the operated limbs as soon as it could be tolerated. The patients were followed up quarterly, with comparison standing full-leg radiographs taken as needed. When the mechanical axis is passed the neutral axis in terms of the middle half of the knee, we removed the implant within 3 months based on the clinical preference and needs of the patients and their parents.

### Radiographic analysis

**Fig. 2:**
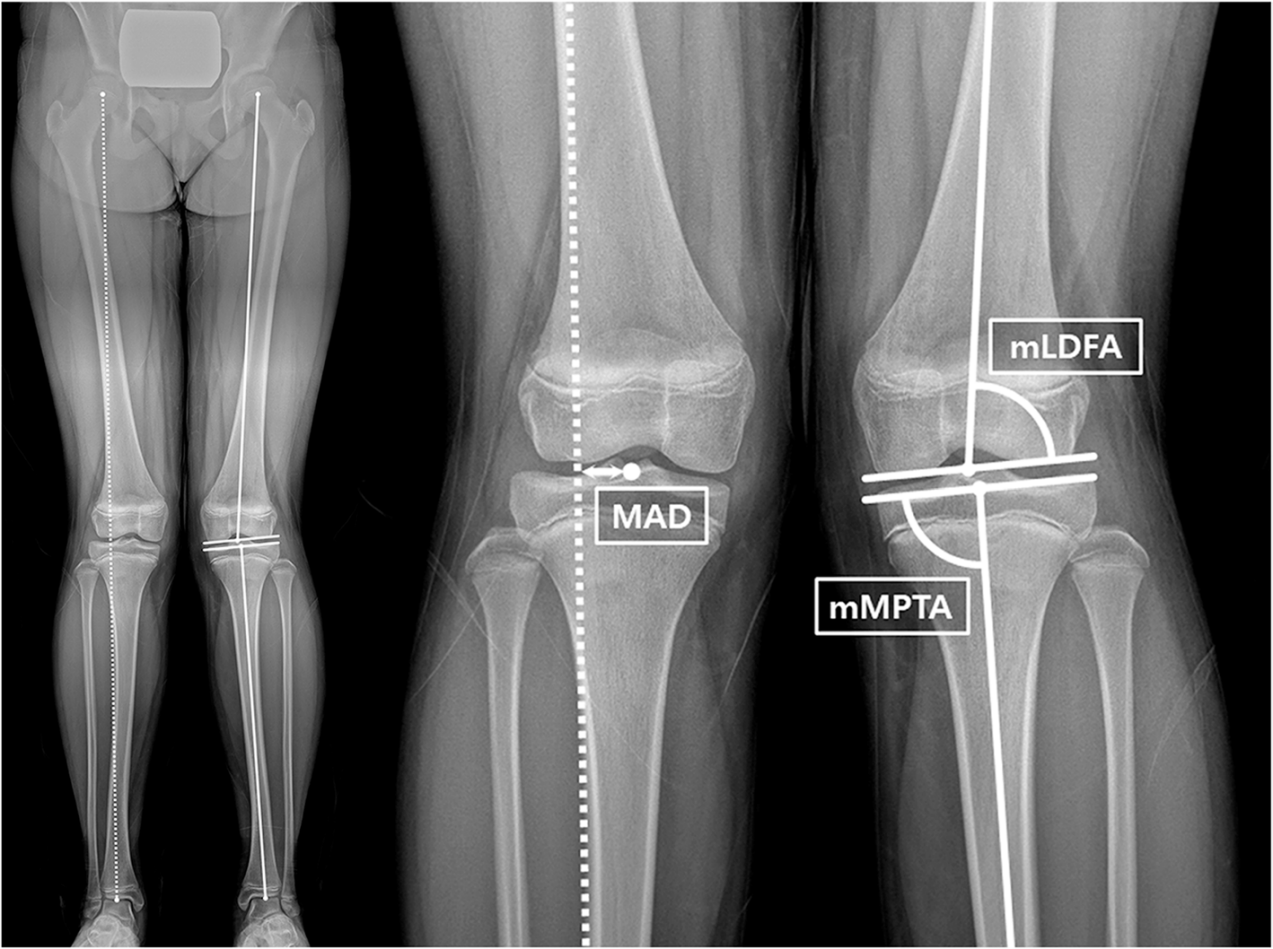
Radiographic measurements of mechanical axis deviation (MAD), mechanical lateral distal femoral angle (mLDFA) and mechanical medial proximal tibial angle (mMPTA).

We measured mechanical axis deviation (MAD) and two mechanical angles (lateral distal femoral angle [mLDFA] and medial proximal tibial angle [mMPTA]) using standing anteroposterior full-leg radiographs with the patella facing forward taken at pre-corrective operation, pre-implant removal, and the final follow-up [19]. The MAD was defined as the distance from the midpoint of the tibial plateau to a line connecting the centers of the hip and ankle joint (Fig. 2). The mLDFA and mMPTA were measured using the mechanical axis of each bone and the corresponding joint line. The correction angle was defined as the difference between the implant insertion and the subsequent removal using the mechanical angle according to the target physis. The correction period refers to the time in months between first and second surgeries, and the correction rate was also calculated as the correction angle divided by the correction period.

Our primary study goals were assessing the correction maintenance of genu valgum after implant removal of guided growth. At the final follow-up, the natural behaviour of each limb was determined as rebound, stable correction, or overcorrection based on the changed degrees of mLDFA or mMPTA after implant removal. Considering the reported median absolute differences of lower-limb parameters [20], a change of > 3° in the direction of the original deformity after implant removal was defined as rebound, and a change of > 3° in the opposite direction was defined as overcorrection. Our secondary study goals were to compare the natural behaviours after implant removal according to the implant character and anatomical site. First, we compared the surgical outcome between PETS and TBP. We evaluated the correction change at the femur and tibia after each PETS and TBP removal.

### Statistical analysis

All statistical analyses were carried out using SPSS version 25.0 (IBM Corp., Armonk, NY), and a *p* < 0.05 was considered statistically significant. Data were assessed for normality using the Shapiro–Wilk’s test. The radiographic parameters were measured and recorded twice by two experienced orthopaedic surgeons, and the intraclass correlation coefficient (ICC) was used to define the interobserver reliability. The ICC was 0.958 (0.953–0.963). To compare the two groups regarding their clinical characteristics and radiologic measurements, an independent *t* test and Mann–Whitney U test were used for continuous variables. Chi-square test and Fisher’s exact test were used according to the expected values to compare categorical variables. We also used Pearson’s correlation analysis and a paired t-test to assess the relationship between the age at implant insertion and the correction rate, and to compare the mechanical angles at two different times, respectively.

## Results

### Guided growth outcome and natural behaviours after implant removal

The mean MAD improved from − 18.8 ± 9.9 mm (range, -46.4 to -4.2 mm) at implant insertion to 11.3 ± 9.4 mm (range, -9.5 to 27.5 mm) at implant removal, and it was noted to have decreased to -0.2 ± 8.9 mm (range, -21.8 to 19.2 mm) at the final follow-up. The implants were removed at an average of 7.7 ± 2.5 months (range, 4 to 15 months) after surgery. There were no postoperative complications, such as surgical wound problems, neurologic deficits, or fractures. At the final follow-up, 39 limbs (53.4%, 25 femurs and 14 tibias) remained stable. The rebound was more common in 22 limbs (30.1%, 16 femurs and six tibias) than the overcorrection in 12 limbs (16.4%, nine femurs and three tibias) (Table 2).

**Table 2:** Correction and natural behaviour after implant removal

Parameters	Values
At implant removal	
Age (years)	11.9 ± 1.8
Mechanical axis deviation (mm)	11.3 ± 9.4
Correction period (months)	7.7 ± 2.5
Weight (kg)	59.8 ± 17.0
Height (cm)	155.4 ± 12.0
BMI (kg/m^2^)	24.2 ± 4.7
At the final follow-up	
Mechanical axis deviation (mm)	-0.2 ± 8.9
Follow-up period after removal (years)	1.9 ± 0.9
Physeal behaviour after removal (no. of limbs)	
Stable	39 (53.4%)
Rebound	22 (30.1%)
Overcorrection	12 (16.4%)

*BMI* body mass index, *PETS* percutaneous epiphysiodesis using a transphyseal screw, *TBP* tension-band plating

Comparing the rebound and non-rebound groups, TBP was more common (81.8% in the rebound group vs. 37.3% in the non-rebound group, *p* < 0.001) and the correction period was longer (*p* = 0.013) in the rebound group (Table 3). There were no significant differences in age, BMI, operation site, correction angle, and correction rate between groups.

**Table 3:** Comparisons between the rebound and non-rebound groups

Parameters	Rebound group	Non-rebound group	*p* value
Age at insertion (years)	11.1 ± 1.9	11.3 ± 1.6	0.388
BMI at insertion (kg/m^2^)	22.8 ± 3.7	24.0 ± 4.7	0.357
Femur vs. Tibia	16 vs. 6	34 vs. 17	0.609
PETS vs. TBP	4 vs. 18	32 vs. 19	< 0.001
Correction period (months)	8.6 ± 2.4	7.2 ± 2.4	0.013
Correction angle (°)	7.4 ± 2.3	6.2 ± 2.6	0.061
Correction rate (° per month)	0.9 ± 0.4	0.9 ± 0.4	0.904
Age at removal (years)	11.9 ± 2.0	11.9 ± 1.7	0.500
BMI at removal (kg/m^2^)	23.2 ± 3.9	24.6 ± 5.0	0.367

*BMI* body mass index, *PETS* percutaneous epiphysiodesis using a transphyseal screw, *TBP* tension-band plating

### Comparison between PETS and TBP

The mean age at both implant insertion (*p* = 0.842) and removal (*p* = 0.881) did not differ significantly between the two groups. No statistically significant differences were also found between the two groups for the mean BMI and correction angle. However, the mean correction period was shorter (*p* < 0.001), and the correction rate was faster (*p* = 0.007) in the PETS group than in the TBP group (Table 4). There was no correlation between the age at surgery and the correction rate (*p* = 0.927, Pearson correlation coefficient = 0.011). Among 36 limbs treated with PETS, the physeal behaviours after implant removal, such as rebound phenomenon and overcorrection, was observed in four (11.1%) and 11 (30.6%) limbs at the final follow-up, respectively. Among 37 limbs with TBP, 18 (48.6%) and one (2.7%), respectively.

**Table 4:** Comparisons between the PETS and TBP groups

Parameters	PETS	TBP	*p* value
Age at insertion (years)	11.2 ± 1.0	11.3 ± 2.2	0.842
BMI at insertion (kg/m^2^)	23.1 ± 4.4	24.1 ± 4.5	0.353
Femur vs. Tibia	23 vs. 13	27 vs. 10	0.404
Correction period (months)	6.3 ± 2.0	9.0 ± 2.1	< 0.001
Correction angle (°)	6.4 ± 3.0	6.7 ± 2.0	0.595
Correction rate (° per month)	1.0 ± 0.4	0.8 ± 0.3	0.007
Age at removal (years)	11.7 ± 1.0	12.0 ± 2.3	0.881
BMI at removal (kg/m^2^)	24.0 ± 4.8	24.3 ± 4.6	0.774

*PETS* percutaneous epiphysiodesis using a transphyseal screw, *TBP* tension-band plating, *BMI* body mass index

### Natural behaviours of femur and tibia after PETS or TBP removal

**Fig. 3:**
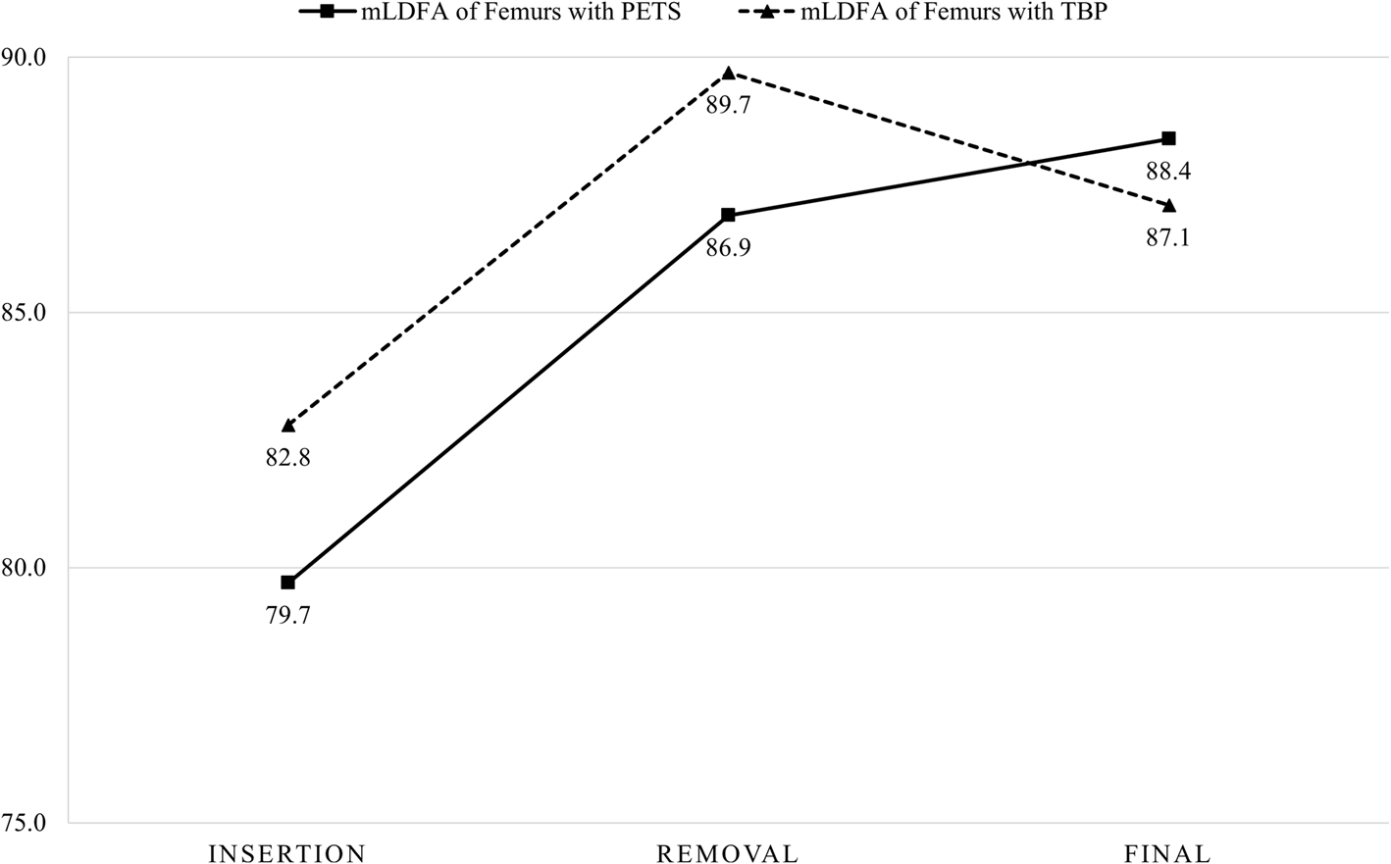
Changes in the mLDFA of distal femurs treated with PETS and TBP.

**Fig. 4:**
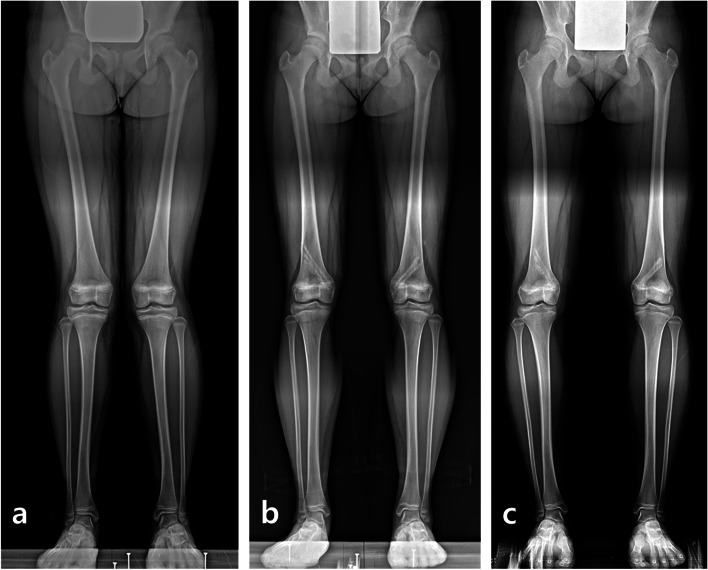
An 11-year-old girl had bilateral genu valgum with a mechanical lateral distal femoral angle (mLDFA) of 82.6° on the right and 80.4° on the left (**a**). Satisfactory alignments were achieved at 6.5 months after percutaneous hemiepiphysiodesis using transphyseal screws, and the alignment was well maintained 2 weeks after implant removal with a mLDFA of 87.5° on the right and 88° on the left (**b**). One year after implant removal, overcorrection of the distal femur was occurred with a mLDFA of 91° on the right and 89.5° on the left, and the mechanical axis deviation increased 22.8 mm and 15.2 mm, respectively (**c**)

Among 50 femurs, 23 treated with PETS were corrected from an average of 79.7 ± 3.4° (range, 68.5 to 86.6°) preoperatively to 86.9 ± 2.8° (range, 82.6 to 91.7°), and other 27 treated with TBP were corrected from 82.8 ± 2.4° (range, 77.8 to 87.3°) to 89.7 ± 2.8° (range, 83.6 to 93.9°) (Fig. 3). However, the mean mLDFA in the PETS group significantly increased to 88.4 ± 2.4° (range, 82.3 to 92.7°) at the final follow-up, indicating overcorrection (mean difference, 1.5 ± 0.6°; 95% confidence interval [CI], 0.1 to 2.8; *p* = 0.031) (Fig. 4), and the mLDFA in the TBP group significantly decreased to 87.1 ± 2.4° (range, 80.6 to 90.3°), indicating rebound (mean difference, -2.6 ± 3.0°; 95% CI, -3.8 to -1.4; *p* < 0.001).

**Fig. 5:**
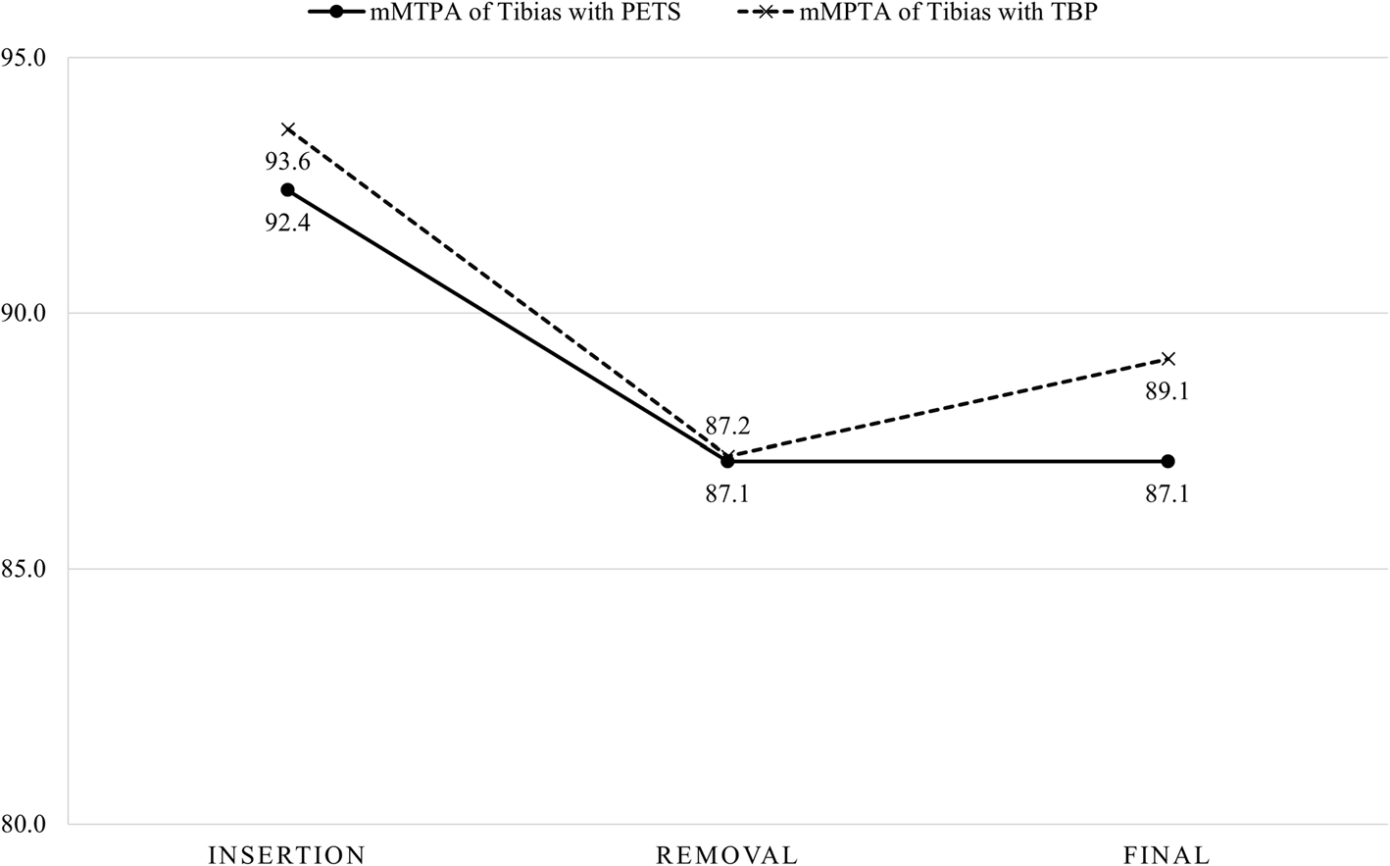
Changes in the mMPTA of proximal tibias treated with PETS and TBP.

In the tibias, the mMPTA of thirteen treated with PETS improved from 92.4 ± 2.9° (range, 89.7 to 97.4°) preoperatively to 87.1 ± 2.3° (range, 83.6 to 90.7°) at implant removal, and from 93.6 ± 2.8° (range, 88.3 to 98.4°) to 87.2 ± 2.3° (range, 83.2 to 91.1°) in ten tibias treated with TBP (Fig. 5). The mean mMPTA was maintained at 87.1 ± 2.5° (range, 83.9 to 92.9°) in the PETS group and increased to 89.1 ± 3.3° (range, 84.2 to 95.5°) in the TBP group at the final follow-up, but there was no significant difference in both groups (*p* = 0.988 and 0.070, respectively).

## Discussion

The most important finding of the present study was that PETS and TBP had different physeal behaviours according to the implant character and anatomical site after guided growth to correct idiopathic genu valgum. Physeal stapling was the first introduced technique for temporary epiphysiodesis [9]; however, the popularity of this technique is decreasing because of several problems, including physeal damage during the procedure, hardware breakage, migration, and extrusion [13, 21, 22]. Instead, transphyseal screw and tension-band plate have been introduced by Metaizeau et al. and Stevens, respectively [10, 11]. Although several studies have compared PETS versus stapling or TBP versus stapling [2, 3, 13, 15, 23], only two studies have compared PETS and TBP in patients with genu valgum [4, 5]. However, none of these described the physeal behaviour such as the stable correction, rebound phenomenon, or overcorrection after implant removal. In our study, 53.4% remained stable after removing the guided growth implant at an average age of 11.9 years; however, 30.1% showed rebound and 16.4% overcorrection.

We could achieve acceptable correction without any serious complications, such as permanent physeal damage or reoperation. PETS led to a faster deformity correction, in concurrence with a previous study [5], and this result was the same in a study for ankle valgus correction [24]. Stapling generally tended to be performed at an earlier age than PETS [2, 15], which is thought to be due to concerns about damage to the growth plate. For the same reason, TBP may be preferred to PETS at an earlier age with high growth remaining, and the younger age can lead to a slow rate of correction compared to the rapid growth rate at adolescents. However, there was no significant difference in age at the time of surgery between the two groups in this study and the number of cases in each group was similar (PETS 36 vs. TBP 37). Additionally, there was no correlation between the age at surgery and the correction rate. Therefore, the time for non-rigid TBP to act as a focal hinge at the perimeter of the physis is thought to be responsible for the difference in the pace of correction between the two implants [4, 11]. After implant removal, approximately half the cases demonstrated stable correction, but the rebound was more common than overcorrection after guided growth. In this predominant rebound group, TBP was more common, and the correction period was longer. The growing potential differs between the distal femur and proximal tibia, so this difference may relate to the correction period. The natural behaviours after implant removal should be separately evaluated according to the femur and tibia considering a different implant character.

Most studies about physeal behaviour after temporary hemiepiphysiodesis have been limited to the rebound phenomenon after TBP or stapling, and the incidence of the rebound has been widely reported between 0 and 60% [15, 17, 18, 25–27]. Such differences are probably due to the heterogeneity of the included patients, especially the etiology of deformity, the potency of growth after implant removal, and the definition of the rebound phenomenon. Our cohort was limited to patients with idiopathic genu valgum and > 1 year of remaining growth after implant removal. The incidences of rebound after PETS and TBP were 11.1 and 48.6% in our series, respectively. Our results showed that the risk factor of the rebound phenomenon was the type of implant. Although there was a difference in the correction period between the two groups, it seems to have originated from the difference in the proportion of implants in each group. Generally, TBP needs a longer correction period, and PETS accounted for 62.7% of the non-rebound group, whereas TBP accounted for 81.8% of the rebound group. TBP had a 4.4 times higher incidence of rebound phenomenon than PETS. Three recent studies focused on the rebound phenomenon reported 55, 42, and 56% of recurrent valgus after TBP, respectively [17, 26, 27], and these values are comparable with our results. A previous study reported a rebound rate of 29% after PETS, which was less than in stapling [2].

Transphyseal fixation of partially threaded screws suppresses the growth plate through direct mechanical compression, and the direct physeal involvement of PETS can make surgeons reluctant to use screws for patients with young age. Brauwer et al. reported a 31% rate of progression of correction after screw removal [2]. Although there were differences in the age at surgery and the definition of progression, we also found an overcorrection rate of 30.6% (11/36 limbs) after PETS. However, the overcorrection after implant removal does not mean permanent physeal arrest. The longest period of screw maintenance was 15 months in our series, but we did not observe any cases with abnormal radiographic findings indicating physeal arrest. Shin et al. also reported no case of physeal arrest after PETS [15]. The compression of the growth plate using PETS may cause a slower return of physeal function compared with TBP, even in the absence of permanent physeal arrest.

The physeal behaviour after implant removal was more pronounced in the distal femur than in the proximal tibia. In both PETS and TBP groups, the mLDFA changed significantly in different directions; overcorrection and rebound, respectively. The changes of mMPTA were in the same direction as the femur depending on the type of implant; however, there was no statistical significance. These findings may be due to different potentials of longitudinal growth or the effect of the fibula as resistant support. The remaining growth after implant removal will contribute significantly to the final coronal alignment, and this suggests that implants should be removed as close to the skeletal maturity as possible. Future studies with guided growth at the other location and younger patients are required.

There were several limitations to this study. First, we selected patients who had remaining growth for more than 12 months after implant removal, which might affect the incidence of rebound. Clinically, the proper guided growth is composed of an accurate prediction of correction and implant removal after skeletal maturation, in which physeal behaviour after implant removal is not expected. The incidence of rebound or overcorrection may be clinically different if we include patients who had undergone guided growth near skeletal maturation. Second, we included only idiopathic genu valgum as a single aetiology, and the small sample size may have weakened the statistical power to detect minor differences. However, single aetiology is more important because of the different effects of the fibula. Another limitation is the lack of randomization related to its retrospective design. The implants were not randomly selected, and the factors considered when choosing an implant could influence the outcomes of guided growth. Further studies with a prospective randomized trial and large cohort including genu varum or various causes of genu valgum are needed to better understand the natural behaviours after implant removal. Lastly, we could not analyse the effect of screw insertion angle in two techniques. The effect of hemiepiphysiodesis may be associated with the convergence angle of two screws in TBP and the angulation of the screw to the physis in PETS [2, 15]. Although we did not measure the inserted angle of each screw, at least three threads of all screws in PETS penetrated the medial quarter of the growth plate, and the two screws in TBP were inserted in parallel [28, 29]. Because all surgeries were performed in the same technique by a single surgeon, we believe that the positions of the screws were similar and that the implant type has a higher effect on the correction process and ensuing physeal behaviour than the minor differences in the position of screws.

## Conclusion

In conclusion, PETS had a faster correction rate for idiopathic genu valgum; however, it tended to be overcorrected in the distal femur after implant removal indicating a slow return of physeal function, and TBP, on the other hand, has a higher probability of rebound. This natural behaviour after guided growth implant removal may be different according to the anatomical site, and the characteristics of each implant must be considered when determining the type of implant and calculating the timing of implant removal, especially in patients who are still growing.

## Data Availability

The datasets used and/or analysed in the current study are available from the corresponding author on reasonable request.

## Abbreviations

PETS: Percutaneous epiphysiodesis using a transphyseal screw
TBP: Tension-band plate
BMI: Body mass index
MAD: Mechanical axis deviation
mLDFA: Mechanical lateral distal femoral angle
mMPTA: Mechanical medial proximal tibial angle
ICC: Intraclass correlation coefficient
CI: Confidence interval
